# Phytochemical Profile and Biological Activity of* Nelumbo nucifera*

**DOI:** 10.1155/2015/789124

**Published:** 2015-12-30

**Authors:** Keshav Raj Paudel, Nisha Panth

**Affiliations:** Department of Pharmacy, School of Health and Allied Science, Pokhara University, P.O. Box 427, Dhungepatan, Kaski, Nepal

## Abstract

*Nelumbo nucifera* Gaertn. (Nymphaeaceae) is a potential aquatic crop grown and consumed throughout Asia. All parts of* N. nucifera* have been used for various medicinal purposes in various systems of medicine including folk medicines, Ayurveda, Chinese traditional medicine, and oriental medicine. Many chemical constituents have been isolated till the date. However, the bioactive constituents of lotus are mainly alkaloids and flavonoids. Traditionally, the whole plant of lotus was used as astringent, emollient, and diuretic. It was used in the treatment of diarrhea, tissue inflammation, and homeostasis. The rhizome extract was used as antidiabetic and anti-inflammatory properties due to the presence of asteroidal triterpenoid. Leaves were used as an effective drug for hematemesis, epistaxis, hemoptysis, hematuria, and metrorrhagia. Flowers were used to treat diarrhea, cholera, fever, and hyperdipsia. In traditional medicine practice, seeds are used in the treatment of tissue inflammation, cancer and skin diseases, leprosy, and poison antidote. Embryo of lotus seeds is used in traditional Chinese medicine as Lian Zi Xin, which primarily helps to overcome nervous disorders, insomnia, and cardiovascular diseases (hypertension and arrhythmia). Nutritional value of lotus is as important as pharmaceutical value. These days' different parts of lotus have been consumed as functional foods. Thus, lotus can be regarded as a potential nutraceutical source.

## 1. Introduction

Traditional medicine or indigenous medicine denotes medical practice developed by local ethnic people via use of natural herbs and mineral. Different place of world has its own history of traditional medicine, for example, ayurvedic medicine originated from Southeast Asia, unani medicine originated in Arab countries/middle east, and acupuncture and traditional Chinese medicine (TCM) originated in China [1–3]. Herbal plants contain secondary metabolite like alkaloids, glycosides, terpene, steroids, flavonoids, tannins, and so forth. Some chemical substances are phenols or their oxygen-substituted derivatives such as tannins while some may contain nitrogen or sulphur that are biologically active and useful for the prevention of disease and treatment of ailment and preserve well-being in humans and animals. Polyphenols exhibit antioxidant activity which lead to many health benefits [4, 5]. Apart from biological activity in human, these chemicals function as plant defense mechanisms against their predator. For human, many of these herbal products and spices serve as useful medicinal source since antiquity [6].

## 2. History

*Nelumbo nucifera* comes under the family Nelumbonaceae, which has various local tribal names (Indian lotus, bean of India, Chinese water lily, and sacred lotus) and several botanical names (*Nelumbium nelumbo, N. speciosa, N. speciosum,* and* Nymphaea nelumbo*). As far as the history is concerned, this beautiful flowering aquatic plant has been honored in the history by three countries: China, India, and Egypt [7–9]. Pictorial representation of the flower can be seen in the art of all of these countries' cultures, symbolizing perfection, purity, and beauty. On the dietary aspect, almost all parts of the plant are used in preparing diverse cuisines depending upon cultures of that place. History reveals that sacred blue lotus (*Nymphaea caerulea*) was distributed and widespread along the Nile river banks [9, 10]. In ancient period, Egyptians worshipped different parts of lotus, widely depicted in their architectural design. Initiation of lotus horticulture in western Europe was introduced by Sir Joseph Banks during 1787 in the form of stove-house water lily whereas these days it is widespread almost everywhere in modern herbal garden. Mostly, lotus plants are popular in Australia pacific, China, India, Korea, and Japan [9, 11].

## 3. Physical Characteristics and Description

*Nelumbo nucifera* is a large aquatic rhizomatous herb consisting of slender, elongated, creeping stem with nodal roots. Lotus is perennial plant with both aerial and floating orbicular leaves. Aerial leaves are cup shaped and floating leaves have flat shape. Its petioles are considerably long and rough with distinct prickles. Flowers vary in color from white to rosy and are pleasantly sweet-scented, solitary, and hermaphrodite. Flower average diameter is 10–25 cm, and it is ovoid and glabrous. Fruit which contain seeds, are black in color, and are hard and ovoid are arranged in whorls; seeds ripened and were released as a result of bending down of pod to the water. Tuberous roots are 8 inches long and 2 inches in diameter. Smooth outer skin of the lotus root is green in color; however, the inner part possesses numerous big air pockets running throughout the length of the tuber assisting for floating in the aquatic system [12, 13].

## 4. Phytochemicals and Constituents

The reported chemical constituents in the different parts of* Nelumbo nucifera* are as shown in the following.

**Figure 1:**
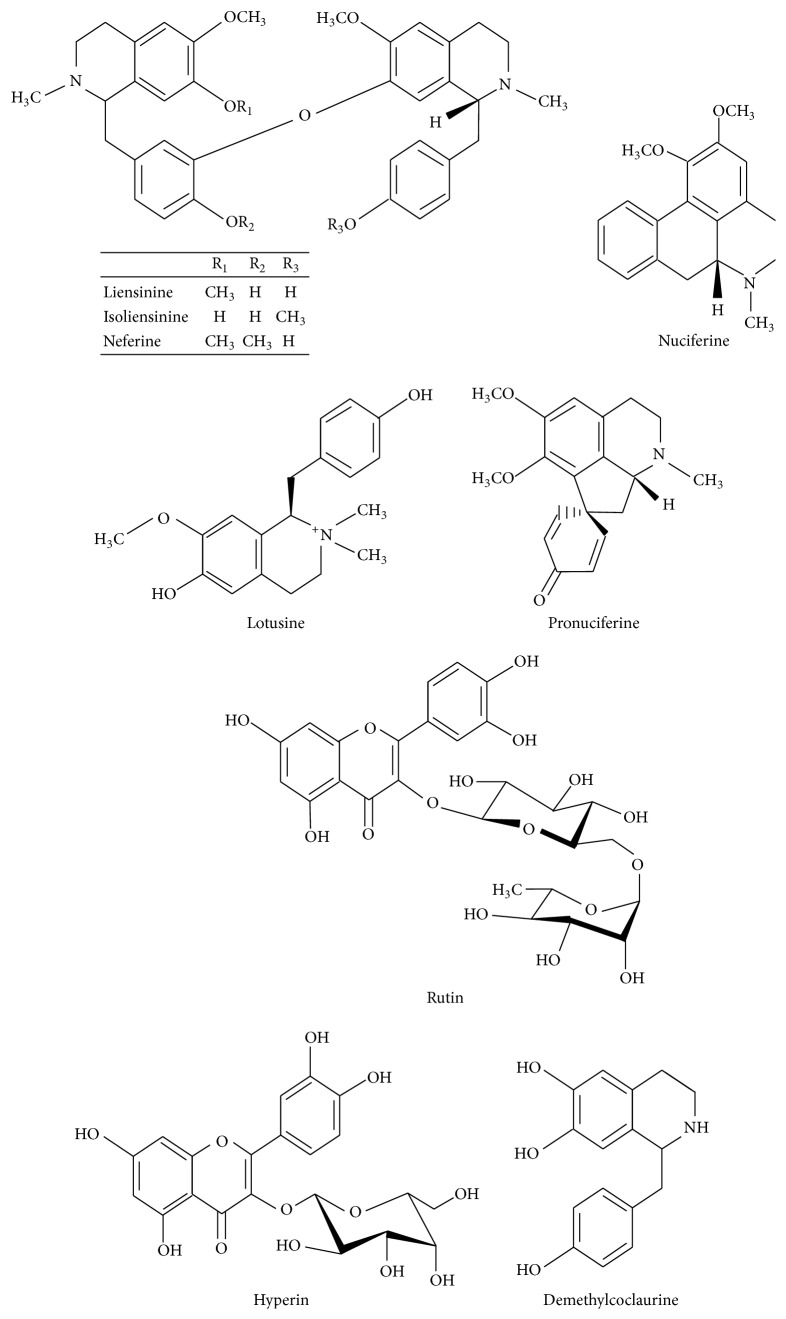
Major chemical constituents present in* Nelumbo nucifera* embryo.

*(1) Embryo*. The phytochemicals reported in embryo of* Nelumbo nucifera* are shown in Figure 1 [14, 15].

**Figure 2:**
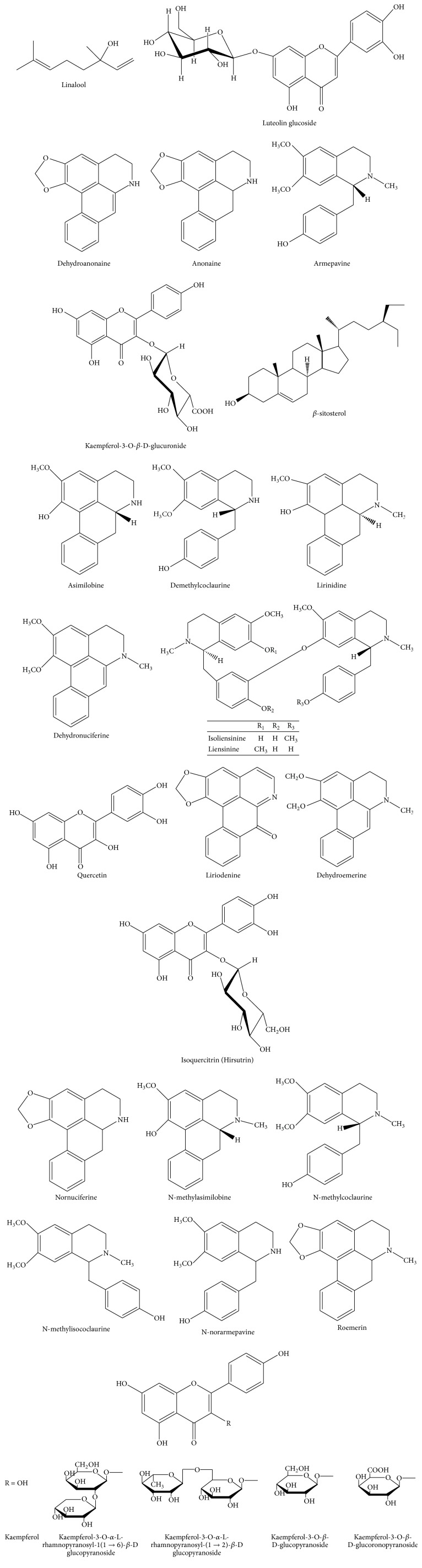
Major chemical constituents present in* Nelumbo nucifera* stamen.

*(2) Stamen*. The phytochemicals reported in stamen of* Nelumbo nucifera* are shown in Figure 2 [12, 16–18].

**Figure 3:**
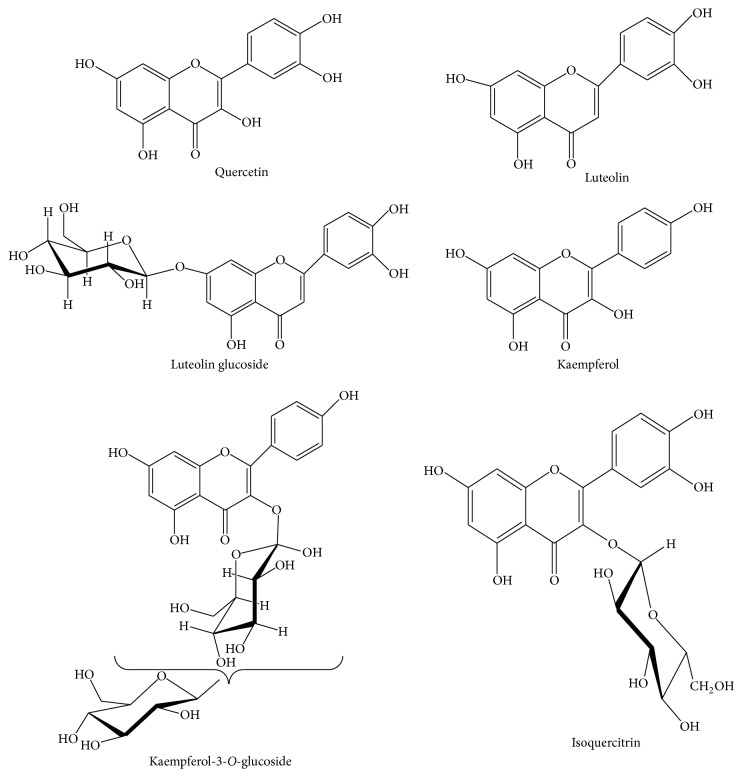
Major chemical constituents present in* Nelumbo nucifera* flower.

*(3) Flower*. The phytochemicals reported in flower of* Nelumbo nucifera* are shown in Figure 3 [16, 19].

**Figure 4:**
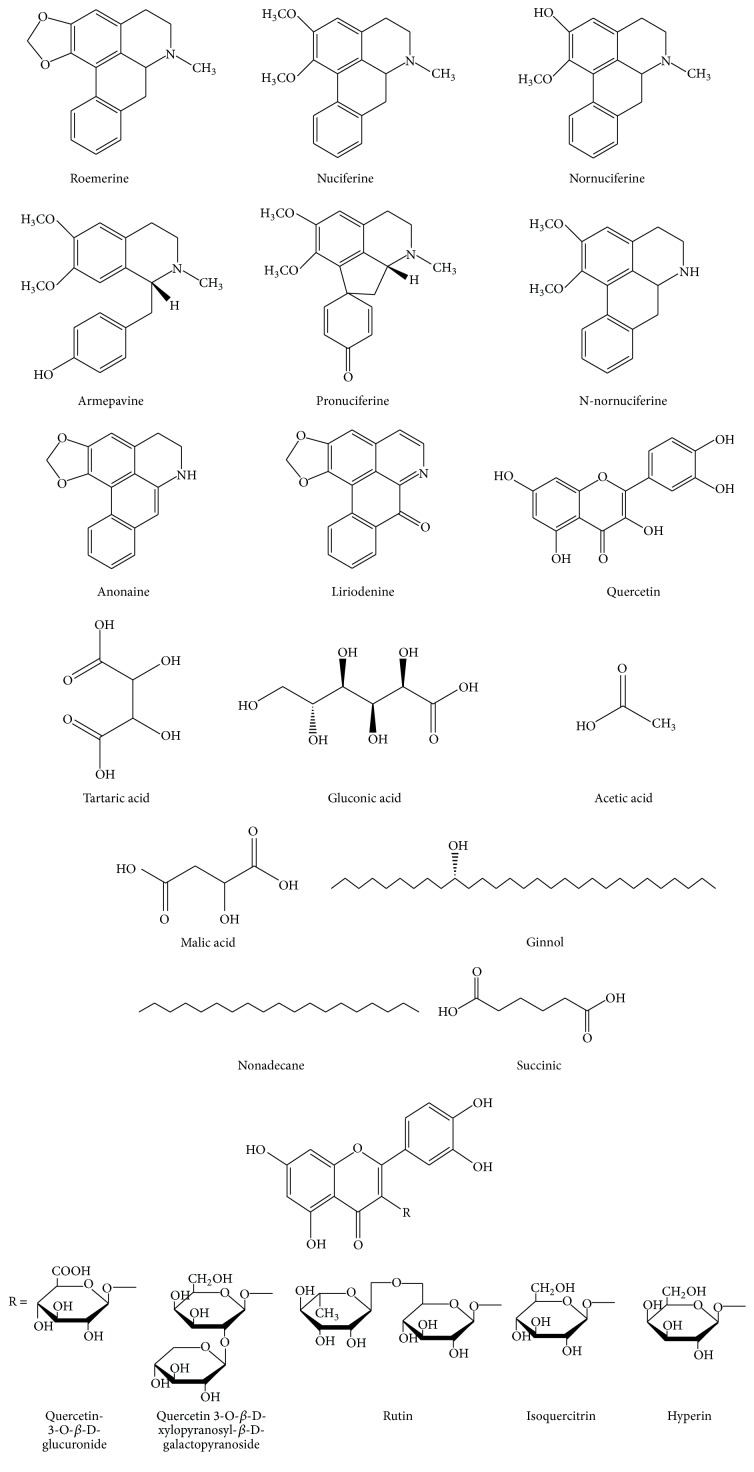
Major chemical constituents present in* Nelumbo nucifera* leaf.

*(4) Leaf*. The phytochemicals reported in leaf of* Nelumbo nucifera* are shown in Figure 4 [12, 16, 19, 20].

**Figure 5:**
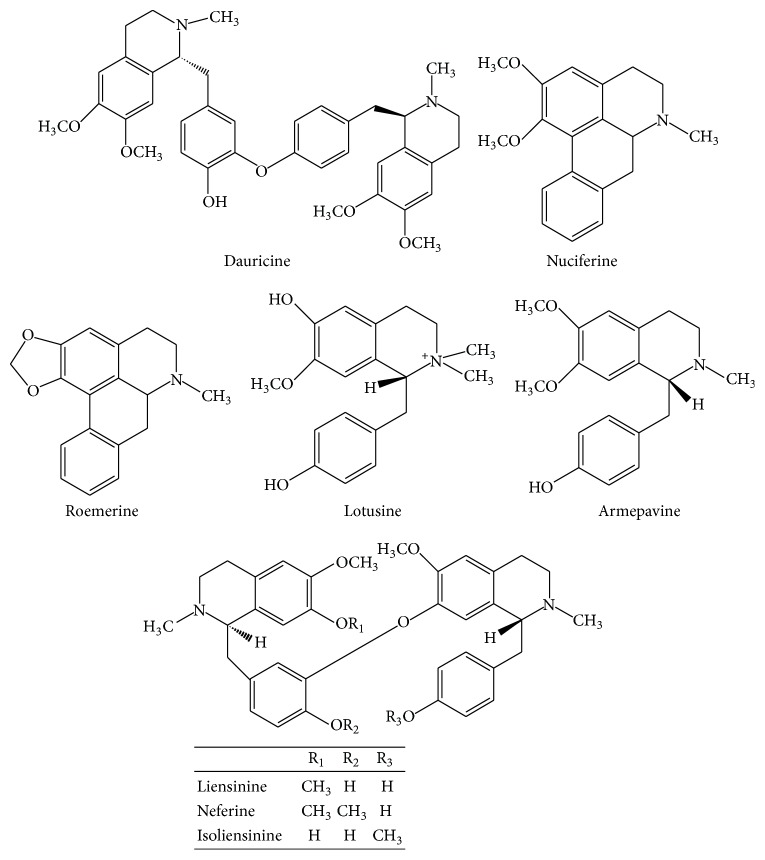
Major chemical constituents present in* Nelumbo nucifera* seeds.

*(5) Seed*. The phytochemicals reported in seed of* Nelumbo nucifera* are shown in Figure 5 [12, 16].

## 5. Reported Pharmacological Activity of* Nelumbo nucifera*

### 5.1. Antioxidants

Hydroalcoholic extract of* Nelumbo nucifera* (HANN) seeds was investigated for antioxidant potency using* in vitro* and* in vivo* models.* In vitro* results showed that HANN exhibited significant DPPH (1,1-diphenyl-2-picrylhydrazyl) free radical scavenging activity with IC_50_ values of 6.12 ± 0.41 *μ*g/mL and nitric oxide (NO) scavenging activity with IC_50_ value of 84.86 ± 3.56 *μ*g/mL. These results were even better than rutin used as standard. In* in vivo* model, HANN administration to Wistar strain rats at a dose of 100 and 200 mg/kg body weight for a time period of 4 days prior to carbon tetrachloride (CCl_4_) administration resulted in a significant dose-dependent raise of superoxide dismutase (SOD) and catalase enzyme level and significant fall in the level of thiobarbituric acid reactive substances (TBARS), as compared to CCl_4_ treated alone (control group) in both liver and kidney. The changes observed at 100 mg/kg body weight treatment were comparable to those observed for tocopherol (50 mg/kg) treatment.* Nelumbo nucifera* seeds contain variety of phytochemical-like saponins, alkaloids, polyphenolics, and carbohydrates supporting significant antioxidant activity of HANN [21]. Lee et al., 2005, studied the antioxidant effect of Korean Traditional Lotus Liquor (Yunyupju) which was made from lotus blossom and leaves. The antioxidant effects were dose-dependent and reached a peak (about 80% inhibition) at a concentration of more than 25 *μ*g of lotus liquor. The liquor exhibited significant DPPH scavenging activities (IC_50_ = 17.9 *μ*g) [22]. Yen et al., 2006, reported the reactive nitrogen species scavenging activity of lotus seed extracts. The potency of lotus seed extracts, namely, water extract (LSWE), ethyl acetate (LSEAE), and hexane (LSHE), to inhibit reactive nitrogen species in macrophage RAW 264.7 cells was investigated. In results, all the extracts of lotus suppress NO production in lipopolysaccharide stimulated RAW 264.7 cells. There was a dose-dependent inhibition of the accumulation of NO upon decomposition of sodium nitroprusside (SNP). In comparison, the potency order was LSEAE > LSWE > LSHE. Further supportive role of lotus extracts to protect DNA breakage in macrophage RAW 264.7 cells promoted by SNP was shown in comet assay. LSWE, LSEAE, and LSHE, at a dose of 0.2 mg/mL, showed 63%, 59%, and 38% inhibition of DNA damage induced by peroxynitrite, respectively. All extracts were found to be peroxynitrite scavengers and thus capable of preventing tyrosine nitration [23]. Ling et al., 2005, extracted the procyanidins from lotus seedpod with Me_2_CO/H_2_O. Among the monomers, dimers, and tetramers of procyanidins, the extract contained high amounts of dimers whereas catechin and epicatechin were the base units. These procyanidins were also investigated for lipid peroxidation, lipoxygenase enzyme activity, and reactive free radical scavenging activity. The superoxide radical scavenging potency of lotus pod procyanidin shows the IC_50_ value of 17.6 mg/L (4-fold less potent as compared to IC_50_ 4.1 mg/L of ascorbic acid). The study also revealed that 0.1% of procyanidins have a significant antioxidant activity in a system of soybean oil, even better than butylated hydroxytoluene (BHT) at a similar concentration [24]. Kim et al., 2014, showed that 80% methanol extract of* Nelumbo nucifera *seed's embryo decreases in ROS production in mouse hippocampal HT22 cells and scavenges DPPH free radical (IC_50_ value as 240.51 *μ*g/mL) and H_2_O_2_ (IC_50_ value as 1769.01 *μ*g/mL) [25].

### 5.2. Antisteroids

The antisteroidogenic activity of* N. nucifera* seed extract in the ovary and testis of the rat was studied by Gupta et al., 1996. In this study, petroleum ether extract fraction was orally given to sexually immature female rats and mature male rats up to 15 days at alternative day. There was a marked delay in sexual maturation in prepubertal female rats as revealed from age of vaginal opening and first estrus (cornified smear) whereas there was reduction in sperm motility and sperm count in male rats. Furthermore, the application of extract resulted in accumulation of ascorbic acid and cholesterol and reduction in *δ*-5-3-*β*-hydroxysteroid dehydrogenase and glucose-6-phosphate dehydrogenase activity in the ovary and testis. Thus, this research explores that the petroleum ether extract can suppress the genesis of steroids in reproductive organs of rats [26]. Similarly, Wethangkaboworn and Munglue, 2014, purpose the 3 hypotheses for inhibitory effect of* N. nucifera* seed extract on male rat sexual behavior to support its antifertility activity: (1) suppression of hypothalamic gonadotropin releasing hormone production, (2) pituitary gonadotropic hormone production, including follicle stimulating hormone (FSH) and luteinizing hormone (LH), or (3) testosterone production [27]. Mutreju et al., 2008, showed that administration of* N. nucifera* to female rats caused estrogen inhibition due to its antiestrogenic nature [28].

### 5.3. Antipyretic Activity

Sinha et al., 2000, reported the antipyretic potential of ethanol extract of lotus stalks on normal body temperature as well as yeast-induced pyrexia using rat as* in vivo* model. Lotus extract, at a dose of 200 mg/kg, markedly decreases the body temperature for a period of 3 hr after administration, while at 400 mg/kg dose it decreases for up to 6 hr. In this yeast model, elevation of body temperature was dose dependently lowered up to 4 hr at both doses by lotus extract and these results were comparable to standard medicine for fever: paracetamol at 150 mg/kg [29]. Similar result was reported by Mukherjee et al., 1996, in yeast-induced pyrexia using rat model [30].

### 5.4. Antiviral Activity

Kashiwada et al., 2005, isolated quercetin 3-O-*β*-D-glucuronide, (+)-1(R)-coclaurine, and (−)-1(S)-norcoclaurine from* Nelumbo nucifera* leaves. The latter two compounds possessed therapeutic activity against HIV with EC_50_ values of 0.8 and <0.8 *μ*g/mL and therapeutic index (TI) values of >125 and >25, respectively, while the first compound was less potent (EC_50_, 2 *μ*g/mL). Other active principles like aporphine, benzylisoquinoline, and bisbenzylisoquinoline alkaloids (liensinine, isoliensinine, and neferine) isolated from leaves and embryo of lotus exhibited potent anti-HIV activities (EC_50_, <0.81 *μ*g/mL; TI of >9.9, >8.6, and >6.5, resp.). An aporphine alkaloid, nuciferine also showed EC_50_, 0.8 *μ*g/mL, and TI of 36 *μ*g/mL [31]. Kuo et al., 2005, reported the antiviral activity of lotus seed ethyl alcohol extract on herpes simplex type 1 (HSV-1). At a dose of 100 *μ*g/mL, ethyl alcohol extract of lotus markedly inhibited HSV-1 replication with IC_50_ of 50.0 *μ*g/mL for replication. Various subfractions of* Nelumbo nucifera* seeds butanol (NN-B) extract were investigated for the HSV-1 inhibitory effects indicating that NN-B-5 out of major nine fractions, NN-B-1 to NN-B-9, had the highest suppresser activity. However, ethyl alcohol extracts obtained from fresh lotus seeds showed anti-HSV-1 activity with IC_50_  62.0 ± 8.9 *μ*g/mL. Furthermore, to get deeper insight of NN-B-5 potency to suppress the acyclovir-resistant HSV-1 replication, the TK HSV-1 strain was targeted and plaque reduction assay was carried out. Results showed 85.9% inhibition of TK HSV-1 replication in HeLa cells by 50 *μ*g/mL of NN-B-5. These results provide evidence that NN-B-5 inhibit the acyclovir-resistant HSV-1 replication [32].

### 5.5. Immunity

Liu et al., 2004, study the effect of ethyl alcohol extracts of lotus in primary human peripheral blood mononuclear cells (PBMC) stimulated by phytohemagglutinin (PHA: a specific mitogen for T lymphocytes) to inhibit the cell proliferation and cytokines production [33]. Liu et al., 2006, also studied the effects of (S)-armepavine from* Nelumbo nucifera* to suppress T cells proliferation. The therapeutic benefit of (S)-armepavine on immune disease, systemic lupus erythematosus (SLE), was investigated on MRL/MpJ-lpr/lpr mice as an* in vivo* model having disease feature similar to human SLE. For this study, (S)-armepavine was treated orally for 6 weeks to MRL/MpJ-lpr/lpr mice and their SLE features were evaluated. The results showed that (S)-armepavine was successful to prevent lymphadenopathy as well as extend the life span of mice. Also, (S)-armepavine treatment resulted in significant decrease of T lymphocyte-mediated cytokines production in dose-dependent manner. Likewise, (S)-armepavine impaired interleukin-2 (IL-2) and interferon (IFN-*γ*) transcripts in human PBMC. This study concluded that (S)-armepavine could be potential therapeutic option as an immunomodulator for the management of SLE [34]. In another study, Liu et al., 2007, revealed the suppressive action on PHA-induced PBMC proliferation and genes expression of IL-2 and IFN-*γ* by (S)-armepavine without direct cytotoxicity [35].

### 5.6. Anti-Inflammatory Activity

Tissue inflammation is harmful response that produces tissue injury and may cause serious diseases such as asthma, atopic dermatitis, and rheumatoid arthritis [36]. There is now convincing evidence that cytokines secreted by T cells such as IL-4, IL-10, and INF-*γ* in response to antigen stimulation play a role in atopic dermatitis, lung inflammation, and asthma [37]. Phytochemical (NN-B-4) identified by a bioassay-based screening procedure from ethanol extract of* Nelumbo nucifera* extracts significantly attenuated proliferation of PMBC induced by phytohemagglutinin, expression of IL-4, IL-10, and INF-*γ*, and cdk-4 gene. NN-B-4 arrest progression of cell cycle of activated PBMC by blocking PBMC from the G1 transition to S phase [32]. Triterpenoid betulinic acid isolated from methanol extract of* Nelumbo nucifera* rhizome was evaluated for the marked anti-inflammatory activity against edema in rat paw caused by carrageenan and serotonin. Methanol extract showed anti-inflammatory activity at doses of 200 and 400 mg/kg p.o. Similarly betulinic acid demonstrated significant anti-inflammatory effect in inflammatory experimental models at doses of 50 mg/kg and 100 mg/kg p.o. Extract and betulinic acid produce similar effect as compared to two potent anti-inflammatory drugs, phenylbutazone and dexamethasone [38].

### 5.7. Diabetes and Complications

The effect of ethanolic extract of* N. nucifera* rhizome was studied in glucose-fed hyperglycemic and streptozotocin-induced diabetic rats. The extract showed improved tolerance action of glucose. Similarly, the potentiating action of extract on the exogenous insulin was seen. The antidiabetic potential of the extract was compared and found to be similar with that of tolbutamide in normal and diseased rat [39]. Such effect could be due to, in part, attenuated absorption of glucose from intestine, as noted, for example, when plant fiber is given orally with glucose [40]. Key enzyme aldose reductase of the polyol pathway has been suggested to play central role in the diabetes and its complications. A methanol extract from* Nelumbo nucifera* stamens revealed inhibitory activity against rat lens aldose reductase. Out of 13 isolated flavonoids from the extract, compounds having 3-O-alpha-1-rhamnopyranosyl-1(1→6)-beta-d-glucopyranoside groups in their carbon ring, including kaempferol 3-O-alpha-1-rhamnopyranosyl-1(1→6)-beta-d-glucopyranoside and isorhamnetin 3-O-alpha-1-rhamnopyranosyl-1(1→6)-beta-d-glucopyranoside, demonstrate highest inhibitory effect of rat lens aldose reductase* in vitro* [41].

### 5.8. Treatment for Erectile Dysfunction

Chen et al. in 2008 investigated the effects of extract neferine on basal concentration of the cyclic adenosine monophosphate and cyclic guanosine monophosphate. Neferine potentiated the cAMP concentration dose dependently; however, this effect was not suppressed by inhibitor of adenylyl cyclase in rabbit corpus cavernosum* in vitro*. Neferine dose dependently increased cAMP accumulation catalyzed by a stimulator of cAMP production, namely, prostaglandin E1 (PGE1). The level of cGMP was not affected by guanylyl cyclase inhibitor and sodium nitroprusside. Neferine enhances the concentration of cAMP in tissue of rabbit corpus cavernosum notably by suppressing activity of phosphodiesterase [42]. In another study, Chen et al. further highlighted treatment of erectile dysfunction of the* in vitro* relaxation mechanisms of neferine on the rabbit corpus cavernosum tissue [43] and* in vitro* effects of neferine on cytosolic free calcium concentration in corpus cavernosum smooth muscle cells of rabbits [44].

### 5.9. Restenosis and Atherosclerosis

Proliferation and expression of matrix metalloproteinase 2 (MMP-2) by smooth muscle cells after percutaneous transluminal coronary angioplasty (PTCA) are responsible for degradation of extracellular matrix (ECM) and migration of smooth muscle cells from tunica media to tunica intima, thereby narrowing the luminal area. Administration of leaf and root extract of* N. nucifera* for 4 weeks significantly suppressed the narrowing of luminal area and stenosis rate in rat model. Similarly, proliferation of vascular smooth muscle cell (VSMC) and expression of MMP-2 in VSMC was dose dependently inhibited by different parts of* N. nucifera* extracts [8]. Similarly, in rabbit model of atherosclerosis induced by high cholesterol diet, leaf extract of* N. nucifera *showed potent antiatherosclerotic activity via inhibition of VSMC proliferation and migration [45] and improved plasma cholesterol level [46]. The active principle of* N. nucifera*, neferine, inhibits angiotensin II-stimulated proliferation in VSMC through heme oxygenase-1 [47] as well as downregulating fractalkine gene expression [48].

### 5.10. Antiaging

Sacred lotus (*Nelumbo nucifera*) seed extract contains antiaging agent that has beneficial effect to reduce symptoms like loss of elasticity, acne, pores, wrinkles, fine lines, blemishes, and so forth. A suitable vehicle possesses the formulations which has potent antiaging agent. It promotes younger looking skin [49]. Mahmood and Akhtar, 2013, study the efficacy of green tea and lotus extract cosmetic formulations for the treatment of facial wrinkles in healthy Asian using a noninvasive device, the Visioscan VC, along with software for surface evaluation of living skin [50] and a noninvasive photometric device (Sebumeter) for the measurement of casual sebum secretions on both sides of the face [51]. Result showed that green tea and lotus combined in multiple emulsions demonstrated a synergistic antiaging effect. The active constituents having antioxidant activity in both herbal plants may possess a beneficial effect on skin surface, thus recommending these plants as the future of new antiaging products.

### 5.11. Antiarrythmia

Phytochemicals, dauricine and neferine, obtained from* N. nucifera* seed have cardiovascular pharmacological effect. Phytochemicals of* N. nucifera* blocked the Na^+^K^+^ and Ca^+2^ cardiac transmembrane current. Notable compound neferine has antiarrhythmic effect and also significantly inhibits aggregation of platelet in rabbits [52]. The antiarrhythmic potency of principle alkaloid from* N. nucifera*, neferine, has been demonstrated in several* in vivo* experimental studies [53]. In a guinea pig papillary muscles and atria, the activity of neferine was investigated on the electrical and mechanical activity. Neferine, at a dose of 0.1 mmol/L, decreased the force of contraction, lowered the amplitude and also *V*_max_ of action potential (AP), and prolonged the action potential duration at 50% (APD50), action potential duration at 90% (APD90), and effective refractory period (ERP). Furthermore, neferine (30 *μ*mol/L) partly antagonized the effect of acetylcholine 10 *μ*mol/L [54]. In guinea pig atria, automaticity inducing effect of adrenaline was attenuated by neferine 30 *μ*mol/L. Similarly, neferine at dose of 30 *μ*mol/L inhibited and shifted dose-effect curve of isoprenaline, an action varied from propranolol action. Also, neferine effect on the Ca^2+^ dose response curve in guinea pigs left atria was studied. Neferine, similar to verapamil, revealed dualistic action in antagonism of Ca^2+^ [55]. Neferine (8 mg/kg, i.v.) electrophysiological effects on ischemic ventricular tachyarrhythmias were studied in both normal and ischemic myocardium by using programmed electrical stimulation (PES) in open chest during 5–8 days after acute myocardial infarction. Neferine was effective in preventing ventricular tachycardias and sudden cardiac death after damage due to myocardial ischemia [56].

### 5.12. Hepatoprotective Activity

Sohn et al., 2003, reported* Nelumbo nucifera* hepatoprotective effect. The protective effects of the ethanol extract of seeds of* Nelumbo nucifera* (ENN) against cytotoxicity induced by CCl_4_ in primary cultured rat hepatocytes were evaluated by the cellular leakage of aspartate transaminase (AST) and cell survival rate. The cellular leakage of AST and the cell death caused by CCl_4_ were significantly inhibited in dose-dependent manners by ENN concentrations between 10 and 500 *μ*g/mL. Similarly, the hepatoprotective effect of ENN against hepatotoxin Aflatoxin B1 (AFB1) was also tested. ENN showed significant hepatoprotective activity at concentrations of both 250 and 500 *μ*g/mL by 74.5% and 94.6%, respectively, as compared with AFB1 controls (48.9%) [57]. Je and Lee investigated the* in vitro* hepatoprotective effect of* Nelumbo nucifera* leaves butanol extract (NNBE) on hepatic damage in cultured hepatocytes induced by H_2_O_2_. The hepatoprotective effect of NNBE was related to the upregulation of various enzymes: superoxide dismutase-1 (SOD-1) by 0.62-fold, catalase (CAT) by 0.42-fold, and heme oxygenase-1 (HO-1) by 2.4-fold. Likewise, pretreatment of NNBE increased the accumulation of nuclear factor erythroid 2-related factor 2 (Nrf2) in nucleus by 8.1-fold signifying that increased SOD-1, CAT, and HO-1 expressions are mediated by Nrf2 [58]. In another study by Yuan et al., ethyl acetate (NUEA) and* n*-butanol (NUBU) extracts of* N. nucifera* leaves were evaluated for hepatoprotective effect on CCl_4_ induced acute liver injury in mice. Except for NUEA group, the levels of aspartate aminotransferase (AST/GOT) and alanine aminotransferase (ALT/GPT) in each treatment group significantly decreased. Moreover, the contents of malondialdehyde (MDA) and the level of SOD in liver of each group were significantly decreased [59]. Likewise, Tang et al. evaluated the mechanisms of hepatoprotective effect of* Nelumbo nucifera* leaves extract (NLE) in* in vivo* model of experimental alcoholic steatohepatitis. The result showed the inhibitory action of NLE on lipid accumulation by altering the levels of triglycerides (TG) and cholesterol (TC) in both plasma and hepatic content analysis. Furthermore, NLE increased the expression of the 5′ adenosine monophosphate-activated protein kinase (p-AMPK/AMPK) ratio and peroxisome proliferator-activated receptor- (PPAR-) *α* responsible for fatty acid oxidation and transport via carnitine palmitoyltransferase-1 (CPT1) and microsomal triglyceride transfer protein (MTP). These results clarify that the NLE can prevent alcoholic steatohepatitis by multiple pathways [60].

### 5.13. Pulmonary Fibrosis

Xiao et al. evaluated the effect of bisbenzylisoquinoline alkaloid isoliensinine isolated from the seed embryo of* Nelumbo nucifera*, on pulmonary fibrosis induced by bleomycin in mice. This study demonstrated that isoliensinine significantly inhibited the hydroxyproline content. Also, isoliensinine subsided histological injury in lungs due to enhancing superoxide dismutase (SOD) activity and malondialdehyde (MDA) level by bleomycin. Moreover, it also inhibits, tumor necrosis factor- (TNF-) *α* and TGF-*β*1 overexpression [61]. Similarly evidence was provided by Zhao et al., 2010, on antifibrosis property of neferine by reversing the decrease in SOD activity, increased in MDA levels and myeloperoxidase activity. Neferine also alleviated bleomycin-induced increase of TNF-*α*, interleukin- (IL-) 6, and endothelin-1 in plasma or in tissue [62]. Niu et al., 2013, showed inhibitory effect of neferine on amiodarone-induced pulmonary fibrosis, due to its potency of anti-inflammation, inhibition of surfactant protein-D (SP-D), and balancing of the increased CD4^+^CD25^+^ regulatory T cells (Tregs) which may modulate Th1/Th2 imbalance by suppressing Th2 response [63].

### 5.14. Antiobesity Activity

Ohkoshi et al., 2007, reported the antiobesity efficacy of active constituents isolated from the leaves of* Nelumbo nucifera* via stimulated lipolysis in mice adipose tissue. Constituent obtained showed antiobesity activity via beta-adrenergic receptor pathway.* Nelumbo nucifera* leaves reduced body weight in A/J mice given high fat diet significantly. Several flavonoids were identified and isolated from NN by fractionation and chromatography. Flavonoids like quercetin, isoquercitrin, catechin, hyperoside, and astragalin showed lipolytic activity in visceral adipose tissue [64]. Ono et al., 2006, outlined the potential pharmacological mechanism of extract isolated from leaves of* N. nucifera* (NNE) in diabetic mice and rats. NNE demonstrated concentration dependent suppression of *α*-amylase, lipase activity, lipid metabolism, and expression of UCP3 mRNA in C2C12 myotubes in mice administrated extract for five weeks. The NNE also exhibited inhibitory activity of *α*-amylase and lipase* in vitro*. Lipase inhibitory activity by NNE (IC_50_, 0.46 mg/mL) was shown to be stronger when compared with *α*-amylase (IC_50_, 0.82 mg/mL). The extract revealed significant suppression in weight gain of body and parametrial adipose tissue. Also extract suppresses concentration of liver triacylglycerol in high fat diet induced obese mice and inhibits UCP3 mRNA expression in skeletal muscle [65]. Similarly, Velusami et al., 2013, study the antiobesity efficacy of methyl alcohol and successive aqueous extracts of* N. nucifera* petals. Parameter studied was effect of those extracts on adipogenesis, adipolysis, lipase, serotonin (5-HT2C), cannabinoid (CNR2), melanocyte concentrating hormone (MCHR1), and melanocortin (MC4R) receptors. Both methyl alcohol and successive aqueous extracts of* N. nucifera* petals inhibited lipid storage in adipocytes and increased lipolysis. Also,* N. nucifera* petal's methyl alcohol extract showed dose-dependent inhibition of lipase activity (IC_50_ value: 47 *μ*g/mL). Furthermore,* N. nucifera* petal extracts possess antagonist and agonist activity on CNR2 and 5-HT2C receptors, respectively. However, it does not show any effect on MCHR1 and MC4R receptors. Overall result summarizes that methyl alcohol extract of* N. nucifera* petals showed better promising activity than successive aqueous extract to control obesity [66].

### 5.15. Anticancer Activity

Various extracts and isolated compound from different parts of* N. nucifera* possess anticancer activity both* in vitro* and* in vivo*. Among the three major alkaloids, isoliensinine possesses the most potent cytotoxic effect, primarily by inducing apoptosis on triple-negative breast cancer cells through ROS generation and p38 MAPK/JNK activation [67].* N. nucifera* markedly suppressed the proliferation of non-small-cell lungs cancer (NSCLC) cells in the presence of nicotine, inhibited Wnt/*β*-catenin signaling activity, promoted the stabilization of Axin, and induced apoptosis.* N. nucifera* decreased the levels of *β*-catenin and its downstream targets including c-myc, cyclin D, and vascular endothelial growth factor- (VEGF-) A.* N. nucifera* also decreased the ratio of B-cell lymphoma 2/Bcl-2-associated X protein (Bcl-2/Bax), which may explain the proapoptosis effect of nuciferine [68]. Out of 15 compounds isolated from leaves from* Nelumbo nucifera*, 7-hydroxydehydronuciferine significantly inhibited the proliferation of melanoma, prostate, and gastric cancer cells [69]. Reversal effect of neferine on multidrug resistance human gastric carcinoma cell line (SGC7901) was studied by Cao et al. SGC7901 and its vincristine- (VCR-) resistant variant (SGC7901/VCR) were treated in the presence or absence of neferine and/or VCR. Neferine reverses multidrug resistance of human gastric carcinoma SGC7901/VCR cells by inhibiting permeability glycoprotein (P-gp) and multidrug resistance-associated protein (MRP) expression in SGC701/VCR cells [70]. In another study, apoptosis inducing effect of neferine was proposed in lung cancer cells (A549 cells). Neferine treatment leads apoptosis by downregulation of nuclear factor kappaB and B-cell lymphoma 2 (Bcl2), upregulation of Bcl-2-associated X protein (Bax) and Bcl-2-associated death promoter (BAD), release of cytochrome C, activation of apoptosis regulator caspase cascade, and DNA fragmentation. Furthermore, neferine caused G1 cell cycle arrest by inducing p53 and its effector protein p21 as well as downregulation of cell cycle regulatory protein cyclin D1 [71]. In addition, neferine possessed growth-inhibitory effect due to cell cycle arrest at G1 on human osteosarcoma cells. It was observed that G1 arrest induction was dependent on p21(WAF1/CIP1); however, it was independent of p53 or RB (retinoblastoma-associated protein). Neferine caused upregulation of p21 due to rise in p21 protein half-life. Researchers examined four kinases that are supposed to affect the stabilization of p21 and found that neferine activated p38 MAPK and JNK. They demonstrated that inhibitor of p38 (SB203580), but not the inhibitor of JNK (SP600125), could decrease p21 upregulation of p21 in response to neferine. Neferine increased phosphorylation of p21 at Ser130 dependent on p38. These results highlighted a direct antitumor action of neferine revealing that neferine possesses cancer-preventive and cancer-therapeutic potential [72].

### 5.16. Nutritional Value

Rhizomes consist of 1.7% protein, 0.1% fat, 9.7% carbohydrate, and 1.1% ash [73]. The taste of rhizome is like beet and exhibits moderate flavor and is used in Chinese cooking [74, 75]. Ogle et al., 2001, reported the use of lotus stem containing 6, 2.4, and 0.2 mg/100 g of calcium, iron, and zinc, respectively, in Vietnamese salad recipes [76]. Lotus leaves are beneficial as natural home remedy for summer heat syndrome and obesity treatment in Asian countries Japan and China [65]. Petals of this plant are sometimes used for garnish and the stamens are used as flavoring agent in the tea [77]. Ibrahim and El-Eraqy in 1996 reported that Egyptian lotus seeds consist of 14.8% crude protein [78]. The green embryos present in the seeds are bitter in taste so they are often eliminated before selling food product to the markets. The seeds can be eaten after popping and can be grounded into flour and eaten raw or used in homemade bread making. Roasted seeds are common substitute of coffee and constitute saponins, phenolics, and carbohydrates in reasonable amount [11, 24].* N. nucifera* seed contains 348.45 cal/100 g energy, 10.6–15.9% protein, 1.93–2.8% crude fat, 2.7% crude fibre, 3.9–4.5% ash, 70–72.17% carbohydrate, and 10.5% moisture [73, 79]. Minerals of lotus seeds contain iron (0.199%), manganese (0.356%), zinc (0.084%), copper (0.0463%), magnesium (9.2%), calcium (22.1%), potassium (28.5%), chromium (0.0042%), and sodium (1%).

### 5.17. Traditional Uses

Traditional knowledge on medicinal uses of lotus plant is demonstrated by many scientific investigations. The whole plant is used as antifungal, antipyretic, emollient, sudorific, diuretic, and cardiotonic. Uses of various parts of the lotus plant are common in tissue inflammation diarrhea treatment and haemostasis [80]. The rhizomes are traditionally used for pectoralgia, leucoderma, pharyngopathy, dysentery, spermatorrhoea, cough, small pox, and diarrhea. In ayurveda, the stem is used as diuretic and anthelmintic and to treat nervous exhaustion, strangury, leprosy, skin disease, and vomiting. Young leaves are mixed with sugar for rectal prolapse therapy. Leaves boiled with goat's milk and* Mimosa pudica* are used as antidiarrheal. Natural remedies obtained from leaf paste are effective in fever and inflammatory skin. Leaves can be used for treatment of metrorrhagia, hematemesis, hemoptysis, hematuria, and epistaxis [81]. Experiment reported treatment of hyperlipidaemia in rodents with lotus leaves [82, 83]. Astringent properties is attributed in leaves to treat strangury, fever, and sweating and as styptic [84]. The flowers and leaves are of importance in treating bleeding disorders. Flowers consumption can promote conception and also are important to treat fever, diarrhea, hyperdipsia, cholera, and hepatopathy. In traditional folk medicines, seeds are used for the treatment of poison antidote and disease of skin and usually prescribed as refrigerant and as diuretic to children [85]. The seeds and fruits are also astringent in nature and can be used for the treatment of various skin diseases, hyperdipsia, halitosis, and menorrhagia [86]. Mixed honey and seed powder is useful in treating cough. Ghee and roots, milk, and gold potentiate strength and virility. Lotus seeds have been used as antimicrobial due to its antimicrobial properties [87, 88]. Lian Zi Xin, Chinese drug, is prepared by using embryo of lotus seed which is useful in insomnia, various cardiovascular diseases (e.g., hypertension and arrhythmia), nervous disorders, and high fevers (with restlessness) [89].

## 6. Conclusions

The perennial aquatic herb,* Nelumbo nucifera,* belonging to family Nymphaeaceae, is gaining its popularity because of its nutraceutical and historical importance. It was used in the treatment of tissue inflammation, cancer, diabetes, skin diseases, bleeding disorders, and cardiovascular diseases as mentioned in the traditional system of medicines. Conducting and documenting the evidence based researches to reveal the possible mechanism of action regarding those effects are today's necessity. Moreover, it will be of economic value if it can be developed as functional food.
